# Study protocol of cost-effectiveness and cost-utility of a biopsychosocial multidisciplinary intervention in the evolution of non-specific sub-acute low back pain in the working population: cluster randomised trial

**DOI:** 10.1186/1471-2474-12-194

**Published:** 2011-08-22

**Authors:** Anna Berenguera, Enriqueta Pujol-Ribera, Teresa Rodriguez-Blanco, Concepció Violan, Marc Casajuana, Nelleke de Kort, Marta Trapero-Bertran

**Affiliations:** 1 Institut Universitari d'Investigació en Atenció Primària Jordi Gol (IDIAP Jordi Gol), Institut Català de la Salut, C/Gran Via de les Corts Catalanes 587 àtic, 08007 Barcelona, Spain; 2 Health Economics Research Group (Herg), Brunel University, Kingston Lane, Uxbridge, Middlesex UB83PH, Great Britain

## Abstract

**Background:**

Low back pain (LBP), with high incidence and prevalence rate, is one of the most common reasons to consult the health system and is responsible for a significant amount of sick leave, leading to high health and social costs. The objective of the study is to assess the cost-effectiveness and cost-utility analysis of a multidisciplinary biopsychosocial educational group intervention (MBEGI) of non-specific sub-acute LBP in comparison with the usual care in the working population recruited in primary healthcare centres.

**Methods/design:**

The study design is a cost-effectiveness and cost-utility analysis of a MBEGI in comparison with the usual care of non-specific sub-acute LBP.

Measures on effectiveness and costs of both interventions will be obtained from a cluster randomised controlled clinical trial carried out in 38 Catalan primary health care centres, enrolling 932 patients between 18 and 65 years old with a diagnosis of non-specific sub-acute LBP. Effectiveness measures are: pharmaceutical treatments, work sick leave (% and duration in days), Roland Morris disability, McGill pain intensity, Fear Avoidance Beliefs (FAB) and Golberg Questionnaires. Utility measures will be calculated from the SF-12. The analysis will be performed from a social perspective. The temporal horizon is at 3 months (change to chronic LBP) and 12 months (evaluate the outcomes at long term).

Assessment of outcomes will be blinded and will follow the intention-to-treat principle.

**Discussion:**

We hope to demonstrate the cost-effectiveness and cost-utility of MBEGI, see an improvement in the patients' quality of life, achieve a reduction in the duration of episodes and the chronicity of non-specific low back pain, and be able to report a decrease in the social costs. If the intervention is cost-effectiveness and cost-utility, it could be applied to Primary Health Care Centres.

**Trial registration:**

ISRCTN: ISRCTN58719694

## Background

Lower back pain (LBP), in any of its forms (acute, subacute, chronic), is one of the principal reasons for consulting the general practitioner (GP). The annual prevalence of LBP is estimated at 14.8% in a study carried out in the Spanish population [1], and these results were confirmed by the 2006 Spanish National health survey [2] and recent data from the Primary Care Information System of Catalonia [3].

LBP has an important impact in the performance of daily tasks and the quality of life of the individuals affected by this pathology. It also has social and family repercussions which are often ignored [1,4]. LBP is one of the six most frequent health problems in developed countries [5,6]. When the LBP diagnosis is established, the minimisation of disability and the cost of labour absenteeism constitutes a shared problem among patients, health professionals, business management, and administration, and makes early intervention necessary to avoid chronic LBP. Some epidemiological studies show that aspects such as psychosocial malaise and fear and avoidance behaviour are associated with a higher risk of developing incapacity in the long term. In patients with these characteristics, a cognitive behavioural intervention has been shown to be effective in reducing this risk [7,8]. Therefore, psychological and social factors associated with the symptoms should be considered because they could affect the level of pain and increase the risk of evolution to chronic LBP [9-12]. Failure to reduce pain represents an important increase in the economic cost to society and reduction in the quality of life of the patient [13,14].

The labour absenteeism caused by LBP is estimated at an average of 21.9 working days lost per illness episode, which represents a cost of 1260 € per worker per year [3]. This cost would be higher if the calculation also took into account "presenteeism" (reduced work productivity), which doubles or triples the cost of labour absenteeism alone [15]. Clinical practice guidelines include scant information about the cost-effectiveness of the treatments, except for those provided by the National Institute for Health and Clinical Excellence (NICE) [16]. In addition, the few studies published have important methodological limitations and are very heterogeneous, which limits the available evidence about the cost effectiveness of LBP interventions [17,18].

Given that the guidelines considered a range of interventions to be effective, it is important to assess the efficiency of treatments versus current intervention throughout the cost-effectiveness analysis [19-21]. As the number of published economic evaluations of interventions for LBP is increasing, it may now be possible to consider evidence of cost-effectiveness when making recommendations about treatment [15,22]. However, no studies have been identified that analyse the cost-effectiveness and cost-utility of multidisciplinary biopsychosocial interventions with respect to habitual practice in primary care.

The objective of the study is to assess from a societal perspective the cost-effectiveness and cost-utility of a multidisciplinary biopsychosocial educational group intervention (MBEGI) in comparison with the usual care of non-specific sub-acute LBP in the working population recruited in Barcelona and its surrounding Primary Health Care Centres (PHCC) since May 2009.

Secondary objectives are to identify and quantify the various costs associated with the disease and their distribution among all of the actors in society.

## Methods/Design

### Study design

Cost-effectiveness and cost-utility analysis of a cluster randomised clinical trial which compares patients with subacute non-specific LBP treated with a MBEGI approach, with a control group receiving only usual clinical care in PHCC. The analysis adopts the societal perspective because, it takes into consideration all other perspectives, will provide disaggregated data, and will make available the most extensive information for use in comparing studies [23].

### Setting (*)

The trial is being conducted in a primary care setting, in 38 PHCCs located in Barcelona, Spain and its surrounding areas. We contact all the PHCCs, present the study to their staff members and invite them to participate.

### Study population

Eligible patients will be identified by the GP or nurses when they consult for a new episode of sub-acute LBP or from searches of electronic clinical records. Patients are informed about the study objectives and those who agree to participate will be given written informed consent to sign.

Patients will be included if the current episode of LBP occurs suddenly after at least 6 months without LBP and lasts between 15 days and 12 weeks [24], and if they do not fulfil any of the exclusion criteria [20].

Furthermore, patients must be between 18 and 65 years old, understand Catalan or Spanish, and be available to participate for at least 12 months. Patients will be excluded if: (a) they are unwilling to participate in the multidisciplinary intervention trial; (b) LBP coexists with cognitive impairment, severe psychiatric disorders such as psychosis, or severe major depression; (c) any other cause of disability impedes answering the various questionnaires; (d) they are pregnant or breast-feeding; (e) they might have anti-inflammatory intolerance or allergy; (f) treatment has been received for physical problems in the preceding 3 months; (g) they have been referred for intensive functional restoration programmes; or (h) they have a confirmed diagnosis of fibromyalgia.

Furthermore, the GP has to ensure that the patient has no red flag signs or symptoms that are frequently associated with specific LBP or potentially severe illnesses [25-28].

### Randomisation

In this study a cluster design is used because the intervention is delivered to groups. To minimise contamination, the unit of randomisation will be the PHCC. Those PHCCs who agree to participate will be randomly allocated to control or intervention groups by a random sequence generated by a computer programme in blocks of random size and prepared before recruitment of the PHCC by an independent statistician who will be blinded to the PHCC identity. The blocking factor was a randomly selected even number (i.e., 4, 6, or 8) and will vary as the recruitment continues. GP or nurses will be informed about their allocation after giving final consent to participation.

To minimise imbalance across intervention groups, randomisation was stratified by percentage of immigrants from developing countries registered in each district. We consider two strata, less than and more than 15% immigrants. This variable is taken as a proxy of socioeconomic level.

### Blinding

During the recruitment, patients who meet the inclusion criteria are allocated to the intervention group corresponding to the centre. To avoid bias, consent to participate is obtained before the allocation. Because of the nature of the intervention, GPs or nurses cannot be blind to patients' allocation. Data analysis will be carried out so that the intervention groups allocated to the patients will be unknown to the analyst.

## Intervention Design

### Control group

Patients allocated to the control group will receive usual clinical care, and individual intervention based on the application of the "Clinical Practice Guidelines in the Pathology of the Lumbar Spine in Adults". These recommendations are published by the Catalan Institute of Health (*Institut Català de la Salut*) [25]. Details are given in Table 1.

**Table 1:** Contents of the clinical guidelines applied in the cluster randomised trial

Clinical Practice Guidelines in the Pathology of the Lumbar Spine in Adults
■ Patient education, give reassuring and positive information about the benign nature of LBP, offer written information including specific advice.
■ Advise avoiding bed-rest and encourage the person to be physically active and continue with normal activities as far as possible.
■ Consider offering a structured physical exercise program tailored to personal preferences
■ Physical exercise should be introduced gently at first (walking, cycling, and swimming) and progressively increased in intensity.
■ Recommend attendance at the "Back School" to those patients who have not resumed their daily tasks, after six weeks.
■ Prescribe pharmacological treatment according to the established guidelines

Notes: LBP = low back pain.

### Intervention group

In addition to the same individual intervention as the control group, patients allocated to the intervention group will receive an educational booklet "The Back Manual" [29] (a transculturally adapted Spanish version of the Back Book) [30] and a biopsychosocial multidisciplinary group intervention.

The group intervention will be carried out by a GP, a nurse, a psychologist and a physiotherapist. The programme consists of 2 sessions of 4 hours duration each and 1 session of 2 hours duration. Each group includes up to 12 participants. Details of the MBEGI intervention and the educational digital video disc (DVD) are included in Tables 2 and 3.

**Table 2:** Components of the biopsychosocial multidisciplinary group intervention

GP + Nurse 2 hours	Objective: Resolve doubts, demystify concepts about LBP and promote adherence to the intervention
	■ Basics on anatomy and biomechanics of the spine
	■ Pain mechanisms
Theory program	■ Causes of pain and predisposing factors
	■ Type of pain, mechanical, inflammatory, and severity
	■ Healthy life habits

Practical program	■ Discuss with the participants the doubts, beliefs and myths about back pain and give positive messages

**Physiotherapist** **4 hours**	**Objective: Provide tools on exercises/postures to avoid the pain and the chronic course and improve quality of life**.

	■ Body posture and its implication in pain
Theory program	■ Ergonomics
	■ Benefits of relative rest

	■ Diaphragmatic breathing exercises as the basis for relaxation, body awareness and postural control.
	■ Pelvic floor/gyration exercises.
Practical program	■ Propioceptive and posture awareness exercises.
	■ Strengthening exercises of the psoas and the posterior chain: Paravertebral muscles, gluteus, ischiotibial muscles.
	■ Strengthening exercises of abdominal muscles, specially the abdominal transversus, gluteus, spinal extensors and scapular muscles.

**Psychologist** **4 hours**	**Objective: Provide participants with cognitive-behavioural therapy techniques**

Theory program	■ Influences of cognitions, emotions and behaviour in pain

	■ Relaxation guidelines and methods
	■ Cognitive restructuring (Modulation of negative thoughts affecting emotions and pain)
	■ Use of attention (Increasing attention focus)
Practical program	■ Assertiveness (improving social relationships)
	■ Problem solving (training in step by step techniques for decision making)
	■ Time organization and reinforcement of reform activities and physical exercise.
	■ Life values (increasing concordance between values and behaviour) ■ Relapse prevention

Notes: GP = general practitioner; LBP = low back pain.

**Table 3:** Contents of the educational Digital Video Disk

Contents of the educational Digital Video Disk
■ Basics on anatomy and biomechanics of the spine
■ Causes and mechanisms of pain
■ Recommendations on dealing with pain and coping with it in daily life
■ A series of stretching, strengthening, and flexibility exercises and methods to promote physical activity
■ Ergonomics applied to daily life (home, work and leisure)
■ Cognitive restructuring (Modulation of negative thoughts affecting emotions and pain)
■ Use of attention (increasing attention focus)
■ Assertiveness (Improving social relationships)
■ Problem solving (training in step by step techniques for decision making)
■ Time organization and reinforcement of reform activities and physical exercise
■ Life values (Increasing concordance between values and behaviour)
■ Relapse prevention

To guarantee the standardisation of the group sessions, only one qualified psychologist and one physiotherapist, both of them with extensive expertise in development of training groups, will apply the intervention in all PHCCs.

#### Outcomes

##### Effectiveness measures

The primary effectiveness measures of the study consist of Roland Morris Disability Questionnaire (RDQ) [31,32] (scale 0-24; lower score indicates lower disability), Mc Gill Pain Questionnaire [33-37] (including VAS 1-10; lower score indicates less pain), and Goldberg questionnaire on anxiety and depression. Duration of days of pain, the reduction of days off work, the reduction of prescription, the duration of pharmacological treatments and recurrent episodes of LBP and the incidence of chronic LBP at 12 months will be measured.

The secondary effectiveness measures are inadequate behaviour and work factors assessed by Fear Avoidance Beliefs Questionnaire (FAB) [38] (scale 0-24; lower score indicates lower fear-avoidance belief), and the Goldberg questionnaire of anxiety and depression [39] (each scale 0-9; lower scores indicates less anxiety or depression). All these questionnaires are validated in Spanish. Patient's assessment of global perceived effect on health will be measured by self-assessment with a Likert 7-point scale [40].

##### Utility measures

The primary utility measure of the study is quality adjusted life years (QALYs), and will be calculated from the SF-12 scores (scale 0-100; lower score indicates poorer quality of life) [32].

##### Other variables

The main independent variable is the intervention arm: MBEGI, or usual clinical care.

Sociodemographic and clinical variables were detailed in the study protocol [21]. Among others, clinical variables are PHCC and hospital emergency visits due to current sub-acute LBP episode; patient compliance with recommendations and treatments; prescribed diagnostic tests; prescribed pharmacological treatment; referrals to other departments; and nonpharmacological treatment measures [41]. Absenteeism will be calculated, and reduced work productivity (presenteeism) will be measured by the Quantity and Quality instrument (QQ). The amount and quality of productivity will be measured on a 10-point numerical rating scale, with 0 representing "nothing" and "very poor quality," respectively, and 10 representing "normal quantity" and "normal quality," respectively [42,43].

##### Other non-pharmacological therapeutic measures and Patient Compliance

The non-pharmacological therapeutic measures are detailed in Table 4.

**Table 4:** Measurements of cost

Measurements of costs					
**Type of cost**	**Costs for**	**Specification**	**Source of resources used**	**Source of costs per 'unit'**	**Cost calculation**

Medical direct costs	Primary Health Care consultations	General Practitioner Practice nurse Physiotherapist Psychologists	Self-reported and comparison the answers by E-cap (electronical clinical records e-cap)	Catalan Institute of Health provider	Number of visits × tariff
	
	Secondary care consultations	Other specialists	Self-reported and comparison by E-cap	Catalan Institute of Health provider	Number of visits × tariff
	
	diagnostic tests	Radiology, magnetic resonance, scanner, electromyogram	Self-reported and comparison by E-cap	Catalan Institute of Health provider	Tests done × tariff
	
	Pharmaceutical treatment	muscle relaxants, analgesics, NSAIDs, corticoids, anxiolytic, Antidepressants and gastric protectors	Self-reported and comparison by E-cap	Standard Pharmaceutical Prices by Consejo General de Colegios Oficiales de Farmacéuticos	Medicines bought × price medicine
	
	Additional medical services	Traumatology, rehabilitation, neurosurgery, other therapies	Self-reported and comparison by E-cap	Catalan Institute of Health provider	Services provided × tariff
	
	Material provided in intervention	leaflet, booklet, CD, DVD	Provider	Provider; production costs	Number of material × price per material
	
	Material used in intervention	mat, postural stool, tennis ball	Provider	Provider; market price	Number of material × price per material

Non medical direct costs	Aid to patients who face disabilities	Aid in household, aid for young children	Self-reported	Patient	hours of aid × price per aid
	
	Additional therapies	Gymnasium, swimming, Yoga/Tai Chi/stretching, other	Self-reported	Patient	Number of months × price per month
		
		Acupuncture, osteopathy, massage, homeopathy, other therapies	Self-reported	Patient	Number of sessions × price per session

Indirect costs	Cost of lost productivity	Absenteeism	Self-reported and comparison by E-cap	Self-reported, profession classification assigned according to Spanish National Institute of Statistics	Days of work sick leave × salary

		Presenteeism	Self-reported	Self-reported, profession classification assigned according to Spanish National Institute of Statistics	reduction percentage × productivity

During follow-up visits (at 3, 6 and 12 months) we will ask the patient about compliance with treatment recommendations and data collection questionnaires will be completed.

##### Measures of resources and costs

The costs analysis will be conducted using a bottom-up approach (bottom-individual; up-societal costs). Average costs will be calculated for each particular type of cost. Costs related to both the MBEGI and the usual care will be collected. The costs of non-specific sub-acute LBP and its treatment consist of direct medical costs and direct and indirect non-medical costs [44]. An overview of the measured costs and the corresponding sources can be found in table 4.

Direct medical costs include those attributable to health care visits for the treatment of LBP: the cost of MBEGI material utilized and distributed to the patients; visits to primary care professionals, to other specialists, and to rehabilitation services; number of complementary tests; and costs of pharmacological treatment and disposable supplies for medical and emergency services use.

These costs will be assessed in accordance with the official rates most recently published in the *Diari Oficial de la Generalitat de Catalunya *(DOGC) for the public health service.

Direct non-medical costs will include home help received as a result of disability related to LBP, patient time directly related to the intervention (time spent in transportation, waiting rooms, and the intervention), non-medical activities (gymnasium, swimming, Yoga/Tai Chi/stretching), and additional therapies (acupuncture, osteopathy, massage, homeopathy).

Indirect non-medical costs include loss of productivity and will be calculated on the basis of time off work (absenteeism) as well as reduced productivity at the workplace (presenteeism). The respondent will be asked to quantify how much work was actually performed during regular hours and the quality of this work as compared with a normal work day [42,43].

Costs will be calculated in euros (€) based on the most actualized prices using the general Spanish consumer price index.

##### Data collection and information sources

All participants will be invited to attend the PHCC for outcome assessments. They will be assessed at the first visit to the PHCC and at 3 months, 6 months, and 12 months after the onset of LBP. Primary data will be collected from the patient by an interview and the electronic clinical records. All outcomes will be measured at the individual level.

The data source of the costs of lost productivity and indirect costs of wages lost to LBP, whether remunerated or not, will be Spain's National Statistics Institute (INE) [44-46], calculated on the basis of the national classification of economic activity [45]. Each participant's profession will be placed into one of the categories of economic activity.

A human capital focus will be used to determine the cost of absenteeism from paid employment. This measure considers the social value of an individual to equal future potential production, measured by the value of anticipated lifetime income. A limitation of this method is it does not take unemployment into account (retirees or those with a disability), although we will correct for this by assigning a value equal to the minimum wage or average salary of their profession [46,47].

##### Sample size

The sample-size calculation is based on change in RDQ at 3 months after onset of LBP. It is recommended that a change of 2 to 3 points on the RDQ should be considered the minimum clinically important change [48]. To allow for the cluster randomisation by PHCC, we assume an intraclass correlation coefficient of 0.1 [49] and a minimum average number of individuals sampled per PHCC of 25. In order to detect a difference of 2.5 points between the two intervention arms with a standard deviation of 5.7 [48,50], an alpha error of 0.05, a beta error of 0.10, and a 20% dropout rate, a sample size of 932 subjects was required, 466 subjects per intervention arm. Therefore, the total number of PHCCs is 38 (19 in each group).

##### Statistical Analysis

The effectiveness of the intervention will be analysed in accordance with CONSORT guidelines, extended to a cluster randomised trial. The analysis will be carried out according to the intention-to-treat principle. Cost, outcomes and use of resources will be reported as mean values with standard deviations for each intervention arm. Comparisons will be done between arms on characteristics of the study population. Mean differences between groups in cost, outcomes and resources, and 95% confidence intervals were calculated. If cost data do not conform to the assumptions for standard statistical tests, the non-parametric bootstrap method will be performed [51].

To address potential biases due to incomplete follow-up, we will analyse patients with complete data at all time points and those with data at any time point, using the multiple imputation approaches to replace missing values. Bias due to non-response will be assessed at each follow-up.

The temporal horizon of the study is 3 months because this is the time of change to a chronic stage and 12 months to evaluate the outcomes at long term.

The discount rate will be 3% [52] that is closest to the actual discount rate.

To establish an important change in individuals, we will contrast the distributions of changes in all the questionnaires and in each assessment, in individuals who change to chronic state and those who do not [48]. We will do the same between those individuals who return to work and those who do not. We will compare individual change scores to the standard error of measurement (SEM).

To detect changes in groups, the intervention effect will be calculated through the effect size for each questionnaire and at each assessment. Effect size will be calculated following the method of Kazis et al [53].

We will evaluate responsiveness of each scale by standardized response mean (SRM) [54] and receiver-operating characteristic method (ROC curve). Since the unit of randomisation is the PHCC, we will use a regression analysis of individual level data using methods for clustered data [55].

To adjust comparisons and to account for cluster randomisation, multilevel linear regression analyses on repeated measures on each of the outcome scales will be used to assess the effect of intervention and to investigate the factors that influence each of the outcome scales at each time point. The possible association between intervention and time will be studied. The PHCC and the individual will be considered as random effects and intervention and time as fixed effects.

The incremental cost-effectiveness ratios were calculated as the difference in mean costs between the MBEGI and the usual care divided by the difference in mean effect, measured in natural unit, between MBEGI and the usual care.

Utilities will be calculated from the SF-12 scores with the corresponding Oxford University algorithm. The QALY will be calculated using these utilities, adjusted by time. The incremental cost-utility ratio will be calculated by dividing the difference in mean total costs between the MBEGI and the usual care by the difference in QALYs [56,57].

To determine the uncertainty surrounding these ratios, 95% confidence intervals for cost-effectiveness ratios will be calculated by the non-parametric bootstrap method [51].

Acceptability curves will be use to determine the probability that the MBEGI will be cost effective compared with usual clinical care at different values of the maximum acceptable ratio.

A deterministic sensitivity analysis will be performed to assess the robustness of the results [58]. The sickness days, GP visits and the number of medications will be measured to assess the impact on costs and cost-effectiveness. The discount rate will be 1% and 5%.

Confidence intervals around point estimates will be reported. The results will be presented in a disaggregated as well as aggregated form.

The significance level of all models will be set at 5%. The SPSS statistical package for Windows, version 19 (SPSS Inc., Chicago, IL) and the Stata/SE version 11·1 for Windows (StataCorp LP) will be used for statistical analysis.

##### Ethical aspects

The study will be conducted according to Guidelines of the Helsinki Declaration and of Good Clinical Research Practice. The project/study protocol has been approved by the Ethical and Clinical Research Committee of IDIAP Jordi Gol, Institute of Research in Primary Health Care.

This trial is registered as Current Controlled Trials (ISRCTN58719694)

More details of the intervention, of patient adherence to the intervention and to non-pharmacological treatment, and the analysis of effectiveness have been published in the protocol for analysis of the effectiveness of the intervention [21].

## Discussion

With this study, we hope to contribute evidence about the cost-effectiveness and cost-utility of MBEGI in reducing episodes of non-specific LBP and the associated social and health costs. This evidence could help health professionals and administrators to make more efficient decisions to address this problem in the primary care setting.

A review of the literature found very few studies of cost-effectiveness and cost-utility related to LBP, and that considered the societal perspective and used a rigorous methodology and a broad sample [59]. On the other hand, the recent review by Catalá-López et al. points out the lack of economic assessment in Spain with respect to musculoskeletal care [60]. The cost-effectiveness and cost-utility analysis of MBEGI proposed in this study, which incorporates most of the relevant costs of LBP, includes a follow-up period that will permit the identification of significant changes and the systematic description of the methods used, will facilitate the interpretation and comparison of the results.

A strength of the study is that the MBEGI has been designed as a pragmatic effectiveness trial. In this type of design, the interventions reflect what may happen in practice and the results are often more generalizable and hence preferable for economic evaluation [44,61].

Information about consumption of resources will be gathered in parallel with the collection of data about the effectiveness of the intervention; therefore, primary data will be obtained in terms of effectiveness and costs to inform the economic evaluation.

The analysis will adopt the social perspective; it is unusual to encounter economic evaluation studies with this perspective, even though LBP is a disease with an important impact on the daily life of the patient as well as the society. Nonetheless, the determination and collection of all of the costs and benefits to the patient, family members, and the National Health Service is a complex task. This study attempts to include the majority of all costs analysed in the studies reviewed. Nonetheless, some costs have been eliminated, such as those attributable to the primary care centre operations, since we could assume that there will be no relevant differences between the treatment and control group in this regard.

It is worth highlighting the inclusion of the concept of presenteeism in estimating labour costs. This concept has not often been applied in calculating the costs of LBP, and seems to have an important impact on those costs [15,62].

The fact that the direct costs of health care will be obtained from the most recent information published in the DOGC will mean that their allocation will be standardized.

We should also note that the participants will be are being recruited from PHCC. Although this aspect could affect the external validity of the study, the National Health Service in Spain provides universal free coverage. More than 70% of the general population visits a primary care centre at least once a year and this percentage increases if we consider longer time periods. In addition, given the size and heterogeneity of the sample and the high number of participating centres, we can assume that the study population represents the Spanish population with this problem, and therefore the results are potentially generalizable.

On the other hand, we have assumed that the usual care attention is to a certain extent homogeneous among the participating GP and nurses. Most of these professionals work for the Catalan Institute of Health and follow the recommendations of the "Clinical Practice Guidelines for lumbar column pathology in adults". Nonetheless, it is very probable that a certain variability exists in their application. In addition, the professionals who participate in research projects tend to be both motivated and more experienced. In any case, we assume that there may be variability, even though this would be similar in both arms of the study.

Another possible limitation is subjects lost to follow-up and/or the non-compliance rate, due to the long duration of the fieldwork. Efforts will be made to minimize these losses with the use of reminders (telephone calls, text messages, and the 6-month interview) that facilitate follow-up and compliance, along with the empathy and communication skills of the interviewers who will be doing the fieldwork.

Despite the 1-year time horizon, our study includes data from more than a natural year, so the possible effect of inflation could slightly modify the prices and values of the different variables. To minimise these effects, we will convert costs and benefits obtained in different years to a base year: the year with the most recently published price levels. In addition, a 3% rate will be applied, which is closest to the actual discount rate.

Another point to consider in the costs estimation is the possible influence of the current economic situation. A review by Degenais et al. [17] showed that the major part of costs derived from LBP are indirect, resulting from the sick leave factor, which has a very important weight in the equation. In addition, due to the current labour uncertainty in our context, workers are more hesitant to request sick leave, which could contribute to underestimation of costs. We will attempt to minimise this impact by including the concept of workforce presenteeism and by the sensitivity analysis.

We also cannot discard the possible effect on costs estimation of the high unemployment rates related to the current economic crisis. Nonetheless, it can be assumed that the level of unemployment would be similar in both study groups, which then would not affect the cost-utility and cost-effectiveness results.

If the intervention is shown to be cost-effective and utility-cost, it could be applied to the primary care population with the expectation of the following results:

■ decreased intensity of LBP

■ decreased duration of the episode and recurrent LBP

■ improved quality of life

■ decreased incidence of chronic LBP

■ decreased days of labour absenteeism and presenteeism

■ reduced pharmacy costs

■ reduced costs to the National Health System in the provision of services due to this pathology

■ Reduced social costs to the patient and his/her family

## Competing interests

The authors declare that they have no competing interests. The effectiveness study was funded by "Fundació La Marató de TV3", a non-profit foundation (grant number 071610), Barcelona, Spain.

The study sponsors have no role in the study design, the collection, analysis, or interpretation of the data, the writing of the report, or the decision to submit the paper for publication.

## Authors' contributions

All authors were responsible for the conception of the project and drafting the first study proposal. AB, EPR, CV, MC, and TRB were involved in writing the manuscript, and all authors critically revised and approved the final manuscript. AB, EPR, TRB, CV, MC, NK, and MTB designed the methodology and TRB designed the statistical analysis. AB, EPR, TRB, MC and CV contributed to the description of the background, designed the questionnaires and made the presentation for the recruitment of the PHCC. All authors read and approved the final manuscript.

## Pre-publication history

The pre-publication history for this paper can be accessed here:

http://www.biomedcentral.com/1471-2474/12/194/prepub

## References

[B1] Humbría Mendiola A Carmona L Peña Sagredo JL Ortiz AM Grupo de Estudio EPISER Impacto poblacional del dolor lumbar en España: resultados del estudio EPISER Rev Esp Reumatol 2002 29 471 478

[B2] Kovacs FM Fernandez C Cordero A Muriel A Gonzalez-Lujan L Gil del Real MT Non-specific low back pain in primary care in the Spanish National Health Service: a prospective study on clinical outcomes and determinants of management BMC Health Serv Res 2006 6 57 10.1186/1472-6963-6-57 16707005 PMC1479820

[B3] Gonzalez Viejo MA Condon Huerta MJ Disability from low back pain in Spain Med Clin (Barc) 2000 114 491 492 10.1016/s0025-7753(00)71342-x 10846653

[B4] Krismer M van TM Strategies for prevention and management of musculoskeletal conditions. Low back pain (non-specific) Best Pract Res Clin Rheumatol 2007 21 77 91 10.1016/j.berh.2006.08.004 17350545

[B5] Lamb SE Hansen Z Lall R Castelnuovo E Withers EJ Nichols V Group cognitive behavioural treatment for low-back pain in primary care: a randomised controlled trial and cost-effectiveness analysis Lancet 2010 375 916 923 10.1016/S0140-6736(09)62164-4 20189241

[B6] van TM Becker A Bekkering T Breen A del Real MT Hutchinson A Chapter 3. European guidelines for the management of acute nonspecific low back pain in primary care Eur Spine J 2006 15 Suppl 2 S169 S191 16550447 10.1007/s00586-006-1071-2 PMC3454540

[B7] Lindstrom I Ohlund C Nachemson A Physical performance, pain, pain behavior and subjective disability in patients with subacute low back pain Scand J Rehabil Med 1995 27 153 160 8602477

[B8] Linton SJ Early identification and intervention in the prevention of musculoskeletal pain Am J Ind Med 2002 41 433 442 10.1002/ajim.10052 12071495

[B9] Krismer M van TM Strategies for prevention and management of musculoskeletal conditions. Low back pain (non-specific) Best Pract Res Clin Rheumatol 2007 21 77 91 10.1016/j.berh.2006.08.004 17350545

[B10] Linton SJ A review of psychological risk factors in back and neck pain Spine 2000 25 1148 1156 10.1097/00007632-200005010-00017 10788861

[B11] Picavet HS Schouten JS Smit HA Prevalence and consequences of low back problems in The Netherlands, working vs non-working population, the MORGEN-Study. Monitoring Project on Risk Factors for Chronic Disease Public Health 1999 113 73 77 10.1016/S0033-3506(99)00122-5 10355306

[B12] Pincus T Burton AK Vogel S Field AP A systematic review of psychological factors as predictors of chronicity/disability in prospective cohorts of low back pain Spine 2002 27 E109 E120 10.1097/00007632-200203010-00017 11880847

[B13] Breivik H Collett B Ventafridda V Cohen R Gallacher D Survey of chronic pain in Europe: prevalence, impact on daily life, and treatment Eur J Pain 2006 10 287 333 10.1016/j.ejpain.2005.06.009 16095934

[B14] Breivik H Borchgrevink PC Allen SM Rosseland LA Romundstad L Hals EK Assessment of pain Br J Anaesth 2008 101 17 24 10.1093/bja/aen103 18487245

[B15] Wieser S Horisberger B Schmidhauser S Eisenring C Brugger U Ruckstuhl A Cost of low back pain in Switzerland in 2005 Eur J Health Econ 2010 10.1007/s10198-010-0258-y PMC3160551 20526649

[B16] Savigny P Watson P Underwood M Early management of persistent non-specific low back pain: summary of NICE guidance BMJ 2009 338 b1805 10.1136/bmj.b1805 19502217

[B17] Dagenais S Caro J Haldeman S A systematic review of low back pain cost of illness studies in the United States and internationally Spine J 2008 8 8 20 10.1016/j.spinee.2007.10.005 18164449

[B18] van der RN Ostelo RW Bekkering GE van Tulder MW de Vet HC Minimal clinically important change for pain intensity, functional status, and general health status in patients with nonspecific low back pain Spine (Phila Pa 1976) 2006 31 578 582 10.1097/01.brs.0000201293.57439.47 16508555

[B19] Airaksinen O Brox JI Cedraschi C Hildebrandt J Klaber-Moffett J Kovacs F Chapter 4. European guidelines for the management of chronic nonspecific low back pain Eur Spine J 2006 15 Suppl 2 S192 S300 16550448 10.1007/s00586-006-1072-1 PMC3454542

[B20] Maetzel A Li L The economic burden of low back pain: a review of studies published between 1996 and 2001 Best Pract Res Clin Rheumatol 2002 16 23 30 10.1053/berh.2001.0204 11987929

[B21] Rodriguez-Blanco T Fernandez-San-Martin I Balague-Corbella M Berenguera A Moix J Montiel-Morillo E Study protocol of effectiveness of a biopsychosocial multidisciplinary intervention in the evolution of non-specific sub-acute low back pain in the working population: cluster randomised trial3 BMC Health Serv Res 2010 10 12 10.1186/1472-6963-10-12 20067619 PMC2820035

[B22] Lin CW Haas M Maher CG Machado LA van Tulder MW Cost-effectiveness of general practice care for low back pain: a systematic review Eur Spine J 2011 10.1007/s00586-010-1675-4 PMC3176699 21203890

[B23] Meltzer MI Introduction to health economics for physicians Lancet 2001 358 993 998 10.1016/S0140-6736(01)06107-4 11583768

[B24] Kovacs FM Abraira V Zamora J Fernandez C The transition from acute to subacute and chronic low back pain: a study based on determinants of quality of life and prediction of chronic disability Spine 2005 30 1786 1792 10.1097/01.brs.0000172159.47152.dc 16094282

[B25] Bordas JM Forcada J García JA Joaniquet FX Pellisé F Mazeres O Guies de pràctica clínica i material docent. Patologia de la columna lumbar en l'adult 2004 Barcelona: Institut Català de la Salut17076930

[B26] Grupo Español de Trabajo del Programa Europeo COST B13 Resumen de las recomendaciones de la Guía de Práctica Clínica para lumbalgia inespecífica 2005

[B27] Kovacs FM Fernandez C Cordero A Muriel A Gonzalez-Lujan L Gil del Real MT Non-specific low back pain in primary care in the Spanish National Health Service: a prospective study on clinical outcomes and determinants of management BMC Health Serv Res 2006 6 57 10.1186/1472-6963-6-57 16707005 PMC1479820

[B28] Rodriguez-Blanco T Fernandez-San-Martin I Balague-Corbella M Berenguera A Moix J Montiel-Morillo E Pujol-Ribera E Study protocol of effectiveness of a biopsychosocial multidisciplinary intervention in the evolution of non-specific sub-acute low back pain in the working population: cluster randomised trial3 BMC Health Serv Res 2010 10 12 10.1186/1472-6963-10-12 20067619 PMC2820035

[B29] Kovacs Fundació El Manual de la Espalda 2002 Palma de Mallorca: Fundació Kovacs

[B30] Burton AK Waddell G Tillotson KM Summerton N Information and advice to patients with back pain can have a positive effect. A randomized controlled trial of a novel educational booklet in primary care Spine 1999 24 2484 2491 10.1097/00007632-199912010-00010 10626311

[B31] Kovacs FM Llobera J Gil del Real MT Abraira V Gestoso M Fernandez C Validation of the spanish version of the Roland-Morris questionnaire Spine 2002 27 538 542 10.1097/00007632-200203010-00016 11880841

[B32] Roland M Morris R A study of the natural history of back pain. Part I: development of a reliable and sensitive measure of disability in low-back pain Spine 1983 8 141 144 10.1097/00007632-198303000-00004 6222486

[B33] Bejarano PF Noriego RD Rodriguez ML Berrio GM Evaluación del dolor: adaptación del cuestionario de Mc Gill Rev Col Anest 1985 13 321 351

[B34] Lahuerta J Smith BA Martinez Lage JM An adaptation of the Mc Gill Pain Questionaire to the Spanish lenguaje Schmerz 1982 3 132 134

[B35] Melzack R The McGill Pain Questionnaire: major properties and scoring methods Pain 1975 1 277 299 10.1016/0304-3959(75)90044-5 1235985

[B36] Ruiz López R Pagerols M Ferrer I The spanish pain questionnaire Pain 1990 5 304S

[B37] Ruiz LR Pagerols BM Ferrer M Collado CA The language of pain Med Clin (Barc) 1991 96 196 2033994

[B38] Kovacs FM Muriel A Medina JM Abraira V Sanchez MD Jauregui JO Psychometric characteristics of the Spanish version of the FAB questionnaire Spine 2006 31 104 110 10.1097/01.brs.0000193912.36742.4f 16395186

[B39] Monton C Perez Echeverria MJ Campos R Garcia CJ Lobo A Anxiety scales and Goldberg's depression: an efficient interview guide for the detection of psychologic distress Aten Primaria 1993 12 345 349 8218816

[B40] Grotle M Brox JI Vollestad NK Concurrent comparison of responsiveness in pain and functional status measurements used for patients with low back pain Spine (Phila Pa 1976) 2004 29 E492 E501 10.1097/01.brs.0000143664.02702.0b 15507789

[B41] Craig CL Marshall AL Sjostrom M Bauman AE Booth ML Ainsworth BE International physical activity questionnaire: 12-country reliability and validity Med Sci Sports Exerc 2003 35 1381 1395 10.1249/01.MSS.0000078924.61453.FB 12900694

[B42] Brooks A Hagen SE Sathyanarayanan S Schultz AB Edington DW Presenteeism: critical issues J Occup Environ Med 2010 52 1055 1067 10.1097/JOM.0b013e3181f475cc 21063183

[B43] Meerding WJ IJzelenberg W Koopmanschap MA Severens JL Burdorf A Health problems lead to considerable productivity loss at work among workers with high physical load jobs J Clin Epidemiol 2005 58 517 523 10.1016/j.jclinepi.2004.06.016 15845339

[B44] Drummond MF Jefferson TO Guidelines for authors and peer reviewers of economic submissions to the BMJ. The BMJ Economic Evaluation Working Party BMJ 1996 313 275 283 8704542 10.1136/bmj.313.7052.275 PMC2351717

[B45] Instituto Nacional de Estadistica (INE) Classificaciones nacionales CNAE 2009 Clasificación nacional de actividades económicas 2010 http://www.ine.es/inebmenu/mnu_clasifica.htm Accessed 1 October 2010

[B46] Koopmanschap MA Rutten FF A practical guide for calculating indirect costs of disease Pharmacoeconomics 1996 10 460 466 10.2165/00019053-199610050-00003 10172868

[B47] Drummond MF Sculpher MJ Torrance GW O'Brien BJ Stoddart GJcost analysis 2005

[B48] Bombardier C Hayden J Beaton DE Minimal clinically important difference. Low back pain: outcome measures J Rheumatol 2001 28 431 438 11246692

[B49] Campbell MK Mollison J Grimshaw JM Cluster trials in implementation research: estimation of intracluster correlation coefficients and sample size Stat Med 2001 20 391 399 10.1002/1097-0258(20010215)20:3<391::AID-SIM800>3.0.CO;2-Z 11180309

[B50] Kovacs F Abraira V Santos S Diaz E Gestoso M Muriel A A comparison of two short education programs for improving low back pain-related disability in the elderly: a cluster randomized controlled trial Spine 2007 32 1053 1059 10.1097/01.brs.0000261556.84266.0f 17471084

[B51] Chaudhary MA Stearns SC Estimating confidence intervals for cost-effectiveness ratios: an example from a randomized trial Stat Med 1996 15 1447 1458 10.1002/(SICI)1097-0258(19960715)15:13<1447::AID-SIM267>3.0.CO;2-V 8841654

[B52] Lopez-Bastida J Oliva-Moreno J Cost of illness and economic evaluation in rare diseases Adv Exp Med Biol 2010 686 273 282 10.1007/978-90-481-9485-8_16 20824451

[B53] Kazis LE Anderson JJ Meenan RF Effect sizes for interpreting changes in health status Med Care 1989 27 S178 S189 10.1097/00005650-198903001-00015 2646488

[B54] Terwee CB Dekker FW Wiersinga WM Prummel MF Bossuyt PM On assessing responsiveness of health-related quality of life instruments: guidelines for instrument evaluation Qual Life Res 2003 12 349 362 10.1023/A:1023499322593 12797708

[B55] Ukoumunne OC Gulliford MC Chinn S Sterne JA Burney PG Methods for evaluating area-wide and organisation-based interventions in health and health care: a systematic review Health Technol Assess 1999 3 iii 92 10982317

[B56] Fox-Rushby J Cairns J The structure of economic evaluation Economic Evaluation 2005 Maidenhead: Open University Press

[B57] Wonderling D Gruen R Black N Economic evalutation Introduction to Health Economics 2005 Maidenhead: Open University Press

[B58] Andronis L Barton P Bryan S Sensitivity analysis in economic evaluation: an audit of NICE current practice and a review of its use and value in decision-making Health Technol Assess 2009 13 iii ix-61 19500484 10.3310/hta13290

[B59] Dagenais S Roffey DM Wai EK Haldeman S Caro J Can cost utility evaluations inform decision making about interventions for low back pain? Spine J 2009 9 944 957 10.1016/j.spinee.2009.07.007 19748833

[B60] Catala-Lopez F Garcia-Altes A varez-Martin E Genova-Maleras R Morant-Ginestar C Parada A Burden of disease and economic evaluation of healthcare interventions: are we investigating what really matters? BMC Health Serv Res 2011 11 75 10.1186/1472-6963-11-75 21489236 PMC3097252

[B61] Ramsey S Willke R Briggs A Brown R Buxton M Chawla A Good research practices for cost-effectiveness analysis alongside clinical trials: the ISPOR RCT-CEA Task Force report Value Health 2005 8 521 533 10.1111/j.1524-4733.2005.00045.x 16176491

[B62] Asche CV Kirkness CS dam-Marx C Fritz JM The societal costs of low back pain: data published between 2001 and 2007 J Pain Palliat Care Pharmacother 2007 21 25 33 18032313

